# Combining MEDLINE and publisher data to create parallel corpora for the automatic translation of biomedical text

**DOI:** 10.1186/1471-2105-14-146

**Published:** 2013-04-30

**Authors:** Antonio Jimeno Yepes, Élise Prieur-Gaston, Aurélie Névéol

**Affiliations:** 1 Lister Hill National Center for Biomedical Communications, U.S. National Library of Medicine, 8600 Rockville Pike, Bethesda, USA; 2 NICTA Victoria Research Lab, Melbourne, VIC, 3010, Australia; 3 Université de Rouen, LITIS EA-4108, 1 rue Thomas Becket, Mont Saint-Aignan, F-76821, France; 4 National Center for Biotechnology Communications, U.S. National Library of Medicine, 8600 Rockville Pike, Bethesda, USA; 5 LIMSI-CNRS, rue John von Neumann, Orsay, F-91400, France

**Keywords:** Multilingual corpus generation, Statistical machine translation, Biomedical domain

## Abstract

**Background:**

Most of the institutional and research information in the biomedical domain is available in the form of English text. Even in countries where English is an official language, such as the United States, language can be a barrier for accessing biomedical information for non-native speakers. Recent progress in machine translation suggests that this technique could help make English texts accessible to speakers of other languages. However, the lack of adequate specialized corpora needed to train statistical models currently limits the quality of automatic translations in the biomedical domain.

**Results:**

We show how a large-sized parallel corpus can automatically be obtained for the biomedical domain, using the MEDLINE database. The corpus generated in this work comprises article titles obtained from MEDLINE and abstract text automatically retrieved from journal websites, which substantially extends the corpora used in previous work. After assessing the quality of the corpus for two language pairs (English/French and English/Spanish) we use the Moses package to train a statistical machine translation model that outperforms previous models for automatic translation of biomedical text.

**Conclusions:**

We have built translation data sets in the biomedical domain that can easily be extended to other languages available in MEDLINE. These sets can successfully be applied to train statistical machine translation models. While further progress should be made by incorporating out-of-domain corpora and domain-specific lexicons, we believe that this work improves the automatic translation of biomedical texts.

## Background

Most institutional and research information in the biomedical domain is available as English text. This is a strong limitation for non-English speakers around the world. Even in countries where English is an official language such as the United States, language can be a barrier for accessing biomedical information due to the high number of non-native English speakers and individuals with low English proficiency. In the United States, Federal regulations require that public-health information be available to individuals with low English proficiency. While some publications do provide patient education hand-outs translated into foreign languages (e.g. [1]), little material is available in languages other than English. In addition, the quality of the translations available for medical documents can be an issue [2]. In the past decade, the issue of access to biomedical information for non-native English speakers has been addressed through cross-language information retrieval [3-5], assuming that the target users had some level of competence in English: queries can be issued in the user’s native language to retrieve documents in English from MEDLINE® or other sources. Related work addressed the development of linguistic resources (e.g. multi-lingual thesauri or dictionaries) that could be used by these systems [6,7] or exploited as useful resources to improve the performance of statistical translation engines applied to specialized domains [8]. More recently, the use of automatic machine translation systems has been investigated to create biomedical documents in foreign languages [5,9-11]. Translation quality remains a major issue, especially when generic translation engines are used. For instance, Zeng-Treiter et al. [9] found that Babel Fish translations [12] of medical record sentences from English into four target languages were incomprehensible and/or incorrect. Wu et al. [11] show that using a specialized biomedical corpus to train a statistical machine translation system improves translation quality significantly. However, they note that large parallel corpora in the biomedical domain are not readily available, which limits the opportunities for quality automatic translations. In this paper, we present a method to obtain large parallel corpora in the biomedical domain relying on MEDLINE data. We apply this method to two language pairs, English/Spanish (EN/ES) and English/French (EN/FR), and evaluate the quality of the resources extracted in two ways: first by direct analysis of the extracted data, and then by applying the data to train statistical machine translation models used to translate biomedical text.

The main contributions of this work are: first, it provides the means of automatically obtaining quality parallel corpora in the biomedical domain and second, it assesses the quality of state-of-the art machine translation obtained for two language pairs in this specialized domain. In addition to reporting the best results to date for machine translation in the biomedical domain, this work outlines directions for future improvements and identifies issues that may be specific to translation resources in the biomedical domain.

## Results

In this section, we show the results obtained during the development of the multi-lingual corpus. Then we show the results obtained from training and testing a statistical machine translation system on this corpus.

### Multi-lingual corpus generation

We present the results of the corpus generation process. We present statistics on MEDLINE for different languages, provide the results of corpus collection from MEDLINE and from the journal websites. Statistics on the final corpus are presented.

#### Multi-lingual resources in MEDLINE

Table 1 presents the availability of MEDLINE resources in languages other than English. It is interesting to note that the availability of abstract and DOI information is not always on par with the number of citations for a given language.

**Table 1:** Availability of foreign language resources in MEDLINE as of October 28, 2011 (top 10 languages in number of citations)

**Language**	**MEDLINE citations**	**With abstract**	**With abstract and DOI**
German	756,385	220,426	59,506
Russian	639,845	196,865	418
French	624,878	176,988	17,270
Japanese	383,419	124,707	1,525
Italian	269,185	58,033	564
Spanish	265,410	92,655	5,439
Chinese	178,146	132,070	958
Polish	160,916	43,486	461
Czech	81,064	16,080	0
Portuguese	75,973	32,607	2,543

In the Methods section, we describe a procedure to recover journal papers given the DOI code from selected publishers from MEDLINE citations that contain an English title and abstract but are not originally written in English. Using this approach, we built a Spanish/English parallel corpus (ENES) by processing 5,439 citations for articles in Spanish collected from the publishers’ website and processed using a Python program based on regular expressions. We were able to extract data from 3,373 of those articles and obtain a 30,000 sentence corpus. Similarly, a French/English corpus (ENFR) was obtained by processing 17,270 citations for articles in French. Data was extracted from 14,817 articles, resulting in a 130,000 sentence corpus. Specific details about each corpus are shown in Table 2. An excerpt from each corpus is shown in Table 3.

**Table 2:** Description of parallel corpora obtained from MEDLINE data

	**ENFR**		**ENES**	
	**English**	**French**	**English**	**Spanish**
Number of citations	14,815	14,817	3,371	3,371
Number of sentences	137,938	130,692	33,167	32,085
Number of words	2,699,851	2,863,638	676,092	760,863

**Table 3:** Excerpts from parallel corpora obtained from MEDLINE data

	
ENFR – PMID 9750586	
ABEN-Partial avulsion of the middle turbinate is an unusual complication of nasotracheal intubation, while minor nasal mucosal trauma is common. We report a case in a 25 year-old healthywoman, diagnosed four years after nasotracheal intubation for removal of wisdom teeth under general anaesthesia, consisting in a unilateral nasal obstruction related to partial avulsion of the middle turbinate.	ABFR-L'avulsion partielle du cornet moyen est une complication inhabituelle de l'intubation nasotrachéale, alors que le traumatisme de la muqueuse nasale est plus fréquent. Nous rapportons le cas d'une patiente de 25 ans, sans antécédent particulier, qui après une intubation nasotrachéale pour extractions dentaires, a présenté une obstruction nasale unilatérale en rapport avec un arrachement du cornet moyen sur toute sa longueur, avec bascule en arrière bloquant la choane.
ENES – PMID 19447450	
ABEN-Bacterial vaginosis is a widely spread health problem with multiple connotations. It has been the subject of many studies and work during decades and it still remains a polemic entity, with contradictory finding. The polymicrobian etiology, unsolved epidemiology, obstetrico-gynecological complications and high recurrence rate following treatment, make this infection a target for researchers. It is not an inflammatory process -yet an immune response exists. In this disorder, vaginal discharge increases, and it is associated with a high risk of developing sexually transmitted diseases.	ABES-La vaginosis bacteriana es un problema de salud ampliamente difundido, con múltiples connotaciones. Ha sido objeto de gran cantidad de estudios y trabajos desde hace décadas y aun en la actualidad sigue siendo una entidad polémica y de resultados contradictorios. La etiología polimicrobiana, la epidemiología no aclarada, las complicaciones obstetroginecológicas y la alta frecuencia de recurrencias tras el tratamiento hacen de esta infección un objetivo para los investigadores. No es un proceso inflamatorio, pero existe una respuesta inmunitaria, cursa con un aumento de flujo vaginal y está asociada a un aumento del riesgo de adquisición de enfermedades de transmisión sexual.

#### Evaluation of parallel corpora extracted from MEDLINE

Table 4 presents the results of the systematic evaluation of the parallel corpora extracted from MEDLINE. It can be seen that the average edit distances are very low for all extracted data types.

**Table 4:** Results of systematic corpus evaluation using edit distance

**Extracted data**	**Total number**	**Average edit distance to MEDLINE data**	**Number with edit distance above 50**
**French (14,817 citations)**			
TIFR	14,817	5.82	325
TIEN	14,815	5.99	347
ABEN	14,089	8.20	1,180
ABFR	14,153	-	-
**Spanish (3,371 citations)**			
TIES	3,371	6.82	70
TIEN	3,371	4.39	14
ABEN	2,961	7.50	148
ABES	2,968	-	-

The manual assessment of abstract pairs showed that when the edit distance [13] was lower than 50 for all extracted fields, the French and Spanish abstracts extracted were adequate translations of English abstracts: 95% of cases for ENFR, 97% for ENES. However, we noticed that even when abstracts are successfully extracted, translation quality may be suboptimal. Specifically, three translation issues could be identified by the evaluation (note that those issues are present in less than 20% of extracted abstracts altogether):

i. One abstract sentence is not translated (for example, see PMID 17904768 for ENFR or PMID 20097448 for ENES)

ii. The extracted abstracts are not a translation of one another - for example, for PMID 16104614, the English abstract (ABEN) is an abridged English version of the French abstract (ABFR).

iii. While abstract content is similar, there are significant phrasing variations for ABEN vs. ABFR (or Spanish abstract (ABES), respectively).

Table 5 shows an analysis of error causes for fields with edit distance above 50. Note that for some citations, more than one field could have an edit distance above 50 so that frequencies may be over the sample size of 100 for the ENFR set and over 218 for the ENES set.

**Table 5:** Analysis of error causes in extracted data

**Extraction**	**Total number**	**Cause**	**Number**
**French**			
Correct	15	No TIFR in MEDLINE	6
		TIFR difference in MEDLINE vs. publisher	9
Incorrect	90	Inverted EN/FR Incomplete title extraction	60
		Keyword extraction instead of title	18
		Incomplete abstract extraction	8
			4
**Spanish**			
Correct	59	Title difference in MEDLINE vs. publisher	9
		No TIES in MEDLINE	49
		Abstract difference in MEDLINE vs. publisher	1
Incorrect	174	Title difference in MEDLINE vs. publisher	7
		Incomplete abstract extraction	66
		Erroneous ABEN extraction	101

For each pair of aligned sentences, *hunalign*[14] provides a score that can be used to filter the aligned sentences and remove sentences with low score that would not positively contribute to the training of the translation system. We have used a subset of 100 sentences for each set English to Spanish and English to French. The aligned sentences have been examined by two judges and a label according to the categories presented in Table 6 has been assigned, mainly to denote the degree of alignment of the sentences. The disagreements have been resolved.

**Table 6:** Manual evaluation of hunalign alignments

	**ENES**		**ENFR**	
**Alignment category**	**Number of sentence pairs**	**Average hunalign score**	**Number of sentence pairs**	**Average hunalign score**
Sentences are unrelated	2	−0.05094	7	0.103413
Some common content, and additional content	1	−0.14269	9	0.284214
ES (resp. FR) has content not covered in EN	11	0.297399	16	0.303225
EN has content not covered in ES (resp. FR)	14	0.571879	14	0.402056
Sentences are aligned	70	0.623229	54	0.497464

Considering the English to Spanish sentences, the alignment is correct in a large number of cases, 70 out of 100. On the other hand, there are 25 in which there is some information that is neither in the foreign nor English sentence and only 3 in which the alignment is not acceptable. There are a total of 17 disagreements out of 100 sentences. Most of them were solved as sentences having more content in the English version or more in the Spanish one. Table 6 shows that most of the sentences are properly aligned with an average score of 0.6232.

Considering the English-to-French sentences, we find that the number of perfectly aligned sentences is 54, which is much lower than the ENES case. There are also around 30 sentences with small differences and 16 sentences in which the alignment is not acceptable. There is a larger disagreement, with 29 sentences out of 100. In many cases, it was found that either the sentences contained the same information or there was a large overlap with some differences between them.

We find that in both scenarios, the threshold for the *hunalign* score could be set around 0.29, since a lower score is related to sentences with additional content or sentences which are completely unrelated.

Table 7 shows the number of remaining sentences after sentence alignment at two *hunalign* score (hr) levels. In the alignment, a large number of sentences are discarded. A large number of sentences are missed but we have preferred to have a more reliable set.

**Table 7:** Alignment of abstract sentences using hunalign

	**ENFR**		**ENES**	
	**English**	**French**	**English**	**Spanish**
**Sentences**	137,938	130,692	33,167	32,085
**Aligned sentences (hr=0.0)**	86,645	86,645	23,316	23,316
**Aligned sentences (hr=0.29)**	52,094	52,094	16,120	16,120

Table 8 shows a case where sentence alignment was not successful. In this example, the Spanish sentences were not properly identified because of the lack of space after the full stop marking the end of the first sentence. In contrast, Table 9 shows a case where sentences were successfully aligned.

**Table 8:** Example of discarded sentences based on their hunalign score

**Hunalign score: 0.105592**	
**EN**	**ES**
The results of prior endoscopic analysis were normal. ~~~ The presence of multiple fundic gland polyps was detected as was their disappearance 6 months after treatment cessation.	No suelen asociar componente displásico.Se describen 4 casos de pacientes en tratamiento crónico con IBP, con endoscopia previa normal, en los que se detectó la presencia de múltiples pólipos de glándulas fúndicas, y se constató su desaparición a los 6 meses tras la supresión del tratamiento.

**Table 9:** Example of sentence properly aligned based on their hunalign score

**Hunalign score: 0.38395**	
**EN**	**ES**
Background and objective: Detection of asymptomatic peripheral arterial disease increases the risk of vascular morbibity and mortality.	Fundamento y objetivo: la detección de arteriopatía periférica silente mediante el índice tobillo-brazo (ITB) incrementa el riesgo de enfermedad y muerte vasculares.

Table 10 presents the statistics on the dataset for each language as well as the partition into different groups (training, tuning and testing) for the SMT experiments.

**Table 10:** Distribution of corpus sentences in translation experiments

	**Training**	**Tuning**	**Testing**
**French**			
**Titles**	458,543	57,317	57,317
**Abstracts (hr=0.0)**	28,882	28,882	28,881
**Abstracts (hr=0.29)**	17,351	17,365	28,881
**Spanish**			
**Titles**	198,512	24,814	24,814
**Abstracts (hr=0.0)**	7,772	7,772	7,772
**Abstracts (hr=0.29)**	5,403	5,418	7,772

For each language pair there are two sets of sentences, the set derived from the titles only and the set made of the abstract sentences we have extracted. The title set has been split into three groups: 80% for training, 10% for tuning and 10% for testing.

The abstract set was significantly smaller; it was split evenly for training, tuning and testing. We believed that the contribution to the training would be small so we preferred to keep a larger portion for tuning and testing the methods.

### Automatic translation of biomedical text using Statistical Translation Models trained using parallel corpora extracted from MEDLINE

The SMT models are evaluated on the test set held out of the title and abstract sentence sets. The performance is measured based on the BLEU metric [15,16]. The BLEU metric is the most popular automatic evaluation metric for machine translation. It counts the matches of n-grams of the candidate translation to n-grams of the reference translation independently of their position. Given the matches, the precision is the ratio of the number of matches in relation to the total number of n-grams generated for that order.

If the output of the decoder is too short, it might obtain a high precision by dropping words. A brevity penalty is computed as shown in the equation below.

(1) $$\mathrm{brevit}y_{\mathrm{penalty}}=\min\left(1,\frac{\mathrm{outpu}t_{\mathrm{length}}}{\mathrm{referenc}e_{\mathrm{length}}}\right)$$

The BLEU metric for a given selection of n is presented in equation 1. *λ*_*i*_ the weights on precision, usually set to 1.

Table 11 shows the BLEU metric for the SMT trained on title sentences and the SMT trained on both title and abstract sentences. The system trained on title sentences performs slightly better compared to that of Wu et al. [11] , which is possibly due to a larger data set or the different selection of training and testing sets. We can see as well that using the abstract sentences, the performance in title translation decreases slightly while the abstract sentence translation improves.

**Table 11:** BLEU metric, training on title corpus and title + abstract sentences corpus decoding results

**Training set**	**Test set**	**EtF**	**FtE**	**EtS**	**StE**
newstest2011	**Titles**	14.09	15.40	20.94	21.19
	**Abs sentences 0.0**	12.00	12.82	18.43	18.77
	**Abs sentences 0.29**	14.19	15.19	19.59	20.29
		**EtF**	**FtE**	**EtS**	**StE**
**Titles**	**Titles**	47.39	47.93	49.93	50.63
	**Abs sentences 0.0**	16.53	18.28	23.36	24.03
	**Abs sentences 0.29**	19.29	21.12	25.00	25.59
		**EtF**	**FtE**	**EtS**	**StE**
**Titles + Abstract Sentences**	**Titles**	47.01	48.05	49.82	50.58
	**Abs sentences 0.0**	20.81	22.54	28.24	28.15
	**Abs sentences 0.29**	24.25	25.78	29.98	30.40

The baseline model (trained on the newstest2011 data set as described in the Methods section) has a lower BLEU value for all the sets, as shown in Table 11. This indicates that training a SMT system on biomedical domain-specific corpora vs. out-of-domain corpora improves the performance of translation for domain-specific texts. This confirms the results obtained by Wu et al. [11] who compared their MEDLINE-trained system to the Google Translate API, used as a baseline translation model not specifically trained to process biomedical text.

In addition to the BLEU metric, we have performed a manual evaluation of the translations. We have randomly selected 100 sentences from each set and have used the measures of fluency and adequacy [15].

Fluency aims to assess both grammatical correctness and idiomatic word choices. It is measured on a 5-point scale where the lowest value corresponds to an incomprehensible sentence and the highest to a flawless sentence. In practice, we established the following guidelines for scoring intermediate sentences: assign a 4 if there was a minor mistake (e.g. agreement error), a 3 if there were several small mistakes, and a 2 for any major mistakes.

Adequacy aims to assess whether the translated sentence carried the same meaning as the original sentence in the source language, considering whether part of the message was lost, added or distorted. Adequacy was measured on a 5-point scale where the lower value indicates that none of the contents of the original sentence was present in the translation, and the higher value indicates that the translated sentence includes all of the original meaning. The following guidelines were used for scoring intermediate sentences: assign a 4 if a small amount of non-essential information is missing (e.g. a date, a non-essential term), a 3 if a fair amount of non-essential information is missing, and a 2 if essential information is missing. This scoring task required native or near-native competence in both languages involved in the scored sets. For this reason, we only had one annotator work on FREN and ENFR (AN) and one annotator work on ESEN and ENES (AJY). Table 12 shows the average value for fluency and adequacy for the four language pairs in our experiments.

**Table 12:** Fluency and adequacy values for the manual evaluation of the translations

	**EtF**	**FtE**	**EtS**	**StE**
**Fluency**	2.96	3.42	3.33	3.46
**Adequacy**	4.21	4.11	3.83	3.98

Table 13 shows examples of scored sentences (in bold) with the corresponding original source sentences. Portions of inadequate text (for fluency) or missing/untranslated content (for adequacy) are underlined.

**Table 13:** Fluency and adequacy examples

**Score**	**Fluency**	**Adequacy**
**1**	Dès lors que la gpa ne contredit aucun de nos droits fondamentaux, on ne peut que souhaiter qu'elle puisse devenir une indication médicale de fiv.	Methods: theoretical sampling and advantages.
	**Whatever the gpa not contredit no fundamental rights, one cannot what souhaiter it may become a medical indication of ivf.**	**Método: la conveniencia muestra.**
**2**	De plus, les individus anxieux manifesteraient une tendance à l’inhibition du partage social des émotions (r=0,26; p=0,05).	L’imagerie montrait un nodule de 1,7 cm du corps pancréatique.
	**Moreover, anxiety individuals manifesteraient a the inhibition of social sharing of emotions (r=0,26; p=0.05).**	**Imaging of 1.7 cm showed a nodule of the pancreatic body.**
**3**	L’imagerie montrait un nodule de 1,7 cm du corps pancréatique.	Cette lésion géodique qui ne semble pas être aussi rare au niveau du carpe, peut être découverte par hasard ou rarement par des douleurs du poignet, exceptionnellement par une fracture.
	**Imaging of 1.7 cm showed a nodule of the pancreatic body.**	**This lesion géodique who does not seem to be also rare in the carp, can be discovered by chance or rarely by pain of the wrist, exceptionally by a fracture.**
**4**	En el presente artículo se describen el diseño y los principales objetivos de un ensayo clínico para evaluar la eficacia y la seguridad del losartán .	Dans les pays industrialisés, la gastroentérite aiguë pédiatrique à rotavirus (geapr) est responsable d’une morbidité élevée.
	**Present article describes the design and the main objectives of a clinical trial to evaluate the efficacy and safety of losartan.**	**In industrialized countries, rotavirus pediatric acute gastroenteritis (geapr) is responsible for a high morbidity.**
**5**	L’évolution a été favorable dans 90% des cas.	L’évolution a été favorable dans 90% des cas.
	**The outcome was favorable in 90% of cases.**	**The outcome was favorable in 90% of cases.**

This table shows examples of scored sentences (in bold) with the corresponding original source sentences. Portions of inadequate text (for fluency) or missing/untranslated content (for adequacy) are underlined. The examples for Adequacy 1 and Fsluency 4 are taken from the ENES corpus while all other examples are from the ENFR corpus.

We can see that in the translations into English, the values for fluency are higher than in the translations from English. Part of this is due to the linguistic characteristics of the languages involved: French and Spanish have strong constraints for gender and number agreement while those constraints are more relaxed with English. Another structural difference is found in the use of articles in English vs. French or Spanish.

The values for fluency are higher for the Spanish vs. French set. This partially correlates to the results obtained in the automatic assessment in which the BLEU metric presented higher values as well. We find that sentence alignment was more straight-forward in the Spanish vs. French set (see Table 6). Even though there is a larger corpus available, the BLEU scores obtained for the translation of abstract sentences in the language pairs involving French are lower than for the translation of abstract sentences involving Spanish. This is consistent with recent work comparing several language pairs on two domain-specific corpora, where BLEU scores for language pairs involving Spanish were about five points higher than the scores for language pairs involving French [17].

The values for adequacy are larger for the French set than for the Spanish set. One of the reasons for this is that in some cases, words are not properly translated in the Spanish set. The French set has a larger number of sentences which covers more vocabulary items.

## Discussion

### Quality of parallel corpora obtained from MEDLINE

Our manual evaluation of error causes (Table 5) shows that language inversion (i.e. labelling an extracted abstract in English ABEN and a French abstract ABFR) is a common error cause. This issue could be addressed by statistically checking that the distribution of extracted text matches that of the language label. Previous work also used URL matching and a comparison of extracted text length as criteria for correct extraction of bitexts [18]. In our case, all texts can be found on the same webpage so that URL analysis is not necessary. However, text length can be useful to identify some erroneous extractions, for instance when keywords are extracted instead of a title.

Interestingly, many cases of “suboptimal” extraction can still provide useful data for some applications such as Statistical Machine Translation, since sentences with lower hunalign scores do not degrade the translation performance (see Table 11).

Contrary to many state-of-the-art parallel corpora used in machine translation studies (e.g. le Hansard, Europarl), professional translators are not involved in the creation of translations in parallel corpora obtained from MEDLINE. In fact, the English text in these corpora is provided by the authors of the papers, i.e. scientists who are neither native English speakers nor trained for translation work. As a result, the English text in MEDLINE parallel corpora may exhibit non-native fluency and present significant semantic distance from the text in the other language. In our study, we observed that these issues were more prevalent in the ENFR corpus vs. ENES. With respect to the use of these corpora in SMT, it raises the question of how the fluency of the bitexts affects machine translation quality. Huck et al. [19] show that using a corpus of automatic translations in addition to human translations provides a (small) improvement of translation quality. However, we are not aware of any studies discussing the influence of various levels of quality for human translations.

### Quality of biomedical text translation

The results shown in Table 6 for “titles” are directly comparable to the work of Wu et al. [11], which previously used MEDLINE titles to train and test a statistical machine translation model. Our results are slightly better and this might simply be due to a larger set of sentences, or a different distribution of the titles in the training vs. the test set. It can also be seen from Table 11 that BLEU scores for title translation are much higher than for abstract sentences. This was expected, since abstract sentences tend to be longer and more complex than titles. Using the title and abstract sentences (vs. titles only) to train the Moses system improves the BLEU score by about 5 points.

Even with a smaller number of example sentences extracted from the abstracts, the translation of abstract sentences improves compared to a system trained solely on title sentences. We can see as well that the larger the set, even with potentially misaligned content, helps improving the performance.

The manual evaluation correlates with the BLEU metric. We see that the manual evaluation confirms that French is more complicated than Spanish in this domain. This result is similar to previous work [17], in which several systems were trained and evaluated on a manually translated set of news articles. In balanced data sets translation results for both French and Spanish to English and vice-versa show mixed results [20].

In addition, the translations into English from either Spanish or French have higher quality due to the strong correlation in gender and number in the Spanish and French languages and to the use of articles in English. Table 14 illustrates the typical caveats found in the automatic translations:

→ failure to translate a word or acronym: e.g. “geapr” in ABEN on line 2, “invasively” in ABFR on line 3;

→ mistranslations: e.g. “incarcerated in the nursery” instead of “attending a daycare” in ABEN on line 2;

→ number and gender agreement: e.g. “sont comparé” instead of “sont comparées” in ABFR on line 3, “progresiva” instead of “progresivo” in ABES on line 5;

→ word order: e.g. “was compared the concentration…” instead of “the concentration was compared” in ABEN on line 4;

→ use of articles: e.g. “is responsible for a high morbidity” instead of “is responsible for high morbidity” in ABEN on line 2.

**Table 14:** Excerpts of abstract sentences translated based on the model trained on title and abstract sentences

**Original sentence**	**Translated sentence**
**ABFR**- dans les pays industrialisés , la gastroentérite aiguë pédiatrique à rotavirus ( geapr ) est responsable d ’ une morbidité élevée , particulièrement chez les enfants de moins de 36 mois gardés en crèche.	**ABEN**- in industrialized countries , rotavirus pediatric acute gastroenteritis ( geapr ) is responsible for a high morbidity , particularly in children less than 36 months *incarcerated* in the nursery.
**ABEN-** the measurements of the arterial blood pressure invasively ( arterial catheter method ) and non invasively ( oscillometric method ) are compared each 12 hours until the catheter is removed.	**ABFR-** la mesure de la pression artérielle *invasively* ( méthode de cathéter artériel ) et non *invasively* ( méthode oscillométrique ) sont comparé chaque 12 heures jusqu' à la sonde est une alternative.
**ABES-** se determinó la concentración de etanol y tóxicos en orina en 66 consultas.	**ABEN**- *was* determined the concentration of ethanol and toxic in urine in 66 cases.
**ABEN-** with progressive lengthening of the muscle , its contractile efficacy increased up to a certain point ( 105% of the length at functional residual capacity ) , after which it began to decline.	**ABES-** alargamiento *progresiva* con del músculo , su eficacia contráctil aumentó hasta un punto de *ciertas* ( 105% de la duración de la capacidad funcional tras residual ) , que se *began* a ocaso.

### Limitations of this work

This work relies on the parallel corpus we obtained from MEDLINE and publisher websites. Therefore its limitations are inherently tied to the corpus features. Because of the method used to obtain the parallel corpus from MEDLINE, the contents of the corpus is limited to articles indexed in the MEDLINE database with a corresponding DOI linking the MEDLINE entry to one of the publisher websites supported by our Python tool. In this work we only addressed two language pairs: English/Spanish and English/French. In addition, the fact that the English portion of the corpus is likely produced by non-native English speakers without formal translation training is to be noted as it differs from other major parallel resources such as Europarl. Another limitation is the fact that sentence alignment was performed automatically, so that some alignment issues remain in the corpus. However, we have made suggestions to overcome this limitation such as defining an alignment score threshold to ensure higher-quality data.

### Directions for future work

#### Obtaining additional parallel corpora

This work can be easily extended to other languages available from MEDLINE. Our experience in developing a tool to extract ENFR text and porting it for extracting ENES data from the same publishers shows that only little work is required. The data sets obtained in this work for the ENFR and ENES language pairs could also be extended by considering additional journals. The problem with this approach is the lack of standard formatting of the articles between journals. This implies developing custom analyzers, one for each journal. As presented in the introduction, there are other resources which could be used to increase the number of sentences in both languages.

In the Results section, we have presented a table with language availability in MEDLINE. The system developed for this research can easily be extended to other languages like German or Russian, which have a larger number of relevant citations in MEDLINE.

#### Improving the results of SMT

We find that abstract sentences are more complex compared to title sentences used in previous work. In addition, French sentences seem to be more difficult to translate compared to Spanish sentences. Spanish translations would benefit from having a larger set to improve the coverage of terms. Current work on out-of-domain data [21] confirms that this would be a path to explore. We would like to explore the use of Europarl corpus, or any corpora available, for this purpose. Another method for improving the quality of translations would be to use a domain-specific lexicon as this was found to be a useful resource for Serbian-English [22]. For the biomedical domain, such lexicons could be obtained from the Unified Medical Language System® (UMLS®) [23]. Finally, translation into English seems easier mainly due to the way articles are used and the less strong correlation between words for gender and number agreement.

## Conclusion

In this paper, we presented a method for obtaining large parallel corpora in the biomedical domain using the MEDLINE database. We show that the quality of the extracted bitexts is high. In turn, they can be used to train Statistical Machine Translation systems and produce the best results to date for machine translation in the biomedical domain. While further progress should be made by incorporating out-of-domain corpora and domain specific lexicons, we believe that this work paves the way for improved automatic translation in the biomedical domain.

## Methods

### Obtaining parallel corpora from MEDLINE

MEDLINE currently indexes about 4,000 journals in the biomedical domain. Although most of them publish articles in English, some also publish articles in other languages. In fact, 22% of the articles indexed in MEDLINE were written in a language other than English. On June 23, 2009, the query *(MEDLINE [sb]) NOT (eng [la])* retrieved 3,847,522 citations, vs. 13,598,239 citations for *(MEDLINE [sb]) AND (eng [la])*. The query *(MEDLINE [sb]) AND (fre [la])* retrieved 601,464 citations, meaning that about 15% of the articles in a language other than English indexed in MEDLINE are in French. However, the detailed distribution of language in MEDLINE shows that English has been increasingly prevalent in recent years [24].

For these articles, MEDLINE citations record the title and abstract in English if they exist, as well as the title in the original foreign language. However, abstracts in foreign languages are not recorded. In recent years, MEDLINE has also been recording DOI (Digital Object Identifier) information for the articles, so that MEDLINE citations can be linked to a webpage where the publishers make additional data available, such as foreign language abstracts.

In this context, it should be possible to obtain a parallel corpus of MEDLINE titles and abstracts by using MEDLINE citations for articles that were originally written in a foreign language and contain DOI information to access publisher pages related to these articles. In this study, we focused on two language pairs as a proof-of-concept to build MEDLINE parallel corpora: English/French and English/Spanish. Due to the heterogeneity of HTML formats, we focused on extracting the following information from two major publishers (EM Consulte (Elsevier) and Science Direct): English title (TIEN), French title (TIFR), Spanish title (TIES), English abstract (ABEN), French abstract (ABFR) and Spanish abstract (ABES). The only information missing from the MEDLINE citations is the foreign language abstracts (ABFR and ABES), but we also extracted information that *was* present in MEDLINE to use as an evaluation proxy for our approach. All publisher data was extracted using a regular-expression Python program, available under [25].

### Evaluation of the elements from the parallel corpora

For a systematic evaluation of the extraction of the French and Spanish abstracts, we made the assumption that, for a given article, if the titles and English abstracts were correctly extracted, it was highly likely that the foreign language abstracts would also be extracted correctly. To assess the extraction of TIEN, TIFR and ABEN, we computed the edit distance between the MEDLINE information and what was extracted, relative to the average length of the strings.

A manual evaluation was also conducted in order to assess the quality of the automatically extracted data. For each corpus, random samples of extracted data were reviewed by the authors (AJY, AN):

1. 100 articles with edit distance lower than 50 for all extracted fields in order to assess whether the extracted abstracts in French or Spanish were an adequate translation of the abstracts in English;

2. 100 articles with edit distance above 50 for at least one extracted field in order to assess the cause of errors.

### Statistical machine translation models

We have used Moses [26] as the toolkit for Statistical Machine Translation (SMT). Moses is a state-of-the-art open-source phrase based SMT. Experiments have been performed following the instructions described in the package [26]. The support packages SRILM and GIZA++ have been installed. SRILM [27] is a package that performs n-gram language models extraction of trigrams. GIZA++ [28] is a statistical machine translation toolkit that is used to train IBM Models 1–5 and an HMM word alignment model.

The experiments with Moses involved three steps: training, tuning and testing. During the training step, Moses learns word-to-word translation and distortion models based on IBM Model 1–5. This model is used to build a phrase table and reordering model. During the tuning step, weights for translation, reordering and language models are learned.

#### SMT corpus preparation

We have two sources for sentences, the titles recovered from MEDLINE and the abstract text collected from the journals.

Abstract text needs to be broken into sentences and aligned to the target/source language translation. Sentence splitting is performed with the Lingua::Sentences tool which has already been used in the context of the Europarl corpus, available in a large number of European languages.

For each citation, sentences in each language pair (ENFR/ENES) are aligned using *hunalign*[14], that aligns bilingual text at the sentence level. Hunalign performs sentence alignment in two steps. First, *hunalign* uses Gale-Church sentence-length information and builds its own dictionary. Then, it performs a second pass using the dictionary and sentence-length information. Once the sentences for each citation have been aligned, a quality assurance process ensures that both sentences are correctly aligned, as described in the Results section.

We have developed an out-of-domain baseline based on the newstest2011 dataset following the instructions and corpora available from [29].

## Competing interests

The authors declare that they have no competing interests.

## Authors’ contributions

AJY participated in the design of the study, carried out the evaluation of the ENES corpus, built the translation models and drafted part of the manuscript. EPG developed the script extracting parallel text elements. AN designed and coordinated the study, prepared the MEDLINE datasets for building the parallel corpora, carried out the evaluation of the ENFR corpus and drafted part of the manuscript. All authors read and approved the final manuscript.
